# Designed Amino Acid Feed in Improvement of Production and Quality Targets of a Therapeutic Monoclonal Antibody

**DOI:** 10.1371/journal.pone.0140597

**Published:** 2015-10-19

**Authors:** Fatemeh Torkashvand, Behrouz Vaziri, Shayan Maleknia, Amir Heydari, Manouchehr Vossoughi, Fatemeh Davami, Fereidoun Mahboudi

**Affiliations:** 1 Biotechnology Research Center, Pasteur Institute of Iran, Tehran, Iran; 2 Process Development Department, Aryogen Biopharma Inc., Alborz, Iran; 3 Department of Chemical & Petroleum Engineering, Biochemical & Bioenvironmental Research Center Sharif University of Technology, Tehran, Iran; Thomas Jefferson University, UNITED STATES

## Abstract

Cell culture feeds optimization is a critical step in process development of pharmaceutical recombinant protein production. Amino acids are the basic supplements of mammalian cell culture feeds with known effect on their growth promotion and productivity. In this study, we reported the implementation of the Plackett-Burman (PB) multifactorial design to screen the effects of amino acids on the growth promotion and productivity of a Chinese hamster ovary DG-44 (CHO-DG44) cell line producing bevacizumab. After this screening, the amino acid combinations were optimized by the response surface methodology (RSM) to determine the most effective concentration in feeds. Through this strategy, the final monoclonal antibody (mAb) titre was enhanced by 70%, compared to the control group. For this particular cell line, aspartic acid, glutamic acid, arginine and glycine had the highest positive effects on the final mAb titre. Simultaneously, the impact of the designed amino acid feed on some critical quality attributes of bevacizumab was examined in the group with highest productivity. The product was analysed for N-glycan profiles, charge variant distribution, and low molecular weight forms. The results showed that the target product quality has been improved using this feeding strategy. It was shown how this strategy could significantly diminish the time and number of experiments in identifying the most effective amino acids and related concentrations in target product enhancement. This model could be successfully applied to other components of culture media and feeds.

## Introduction

Recent progress in cell culture technology for recombinant CHO cells has led to substantial enhancements in target protein’s production [1, 2]. This advancement in the production yields is mostly due to the extension of stable high producers through vector design and host cell engineering methods, as well as medium optimization and process development [3–5].

The formulation of media and feeds is an important phase of the process development. Manufacturers spend extensive efforts and time on the optimization of culture media and feeds as a basic development process for each cell line [4, 6]. However, no generic procedure exists for cell culture medium and feed optimization, and reports on the optimization of culture media or feeding strategies for CHO cell cultures are limited in number. A valuable start to optimizing the medium or feed is to focus on basic groups of ingredients composing mammalian cell culture media. Traditional optimization methods, such as the titration of single components, are reliable, but can be labour intensive and time consuming [7–9].

Design Of Experiment (DOE) methods are useful in some optimization steps for decreasing the number of experiments [4]. In fact, any strategy for decreasing the large number of components in media or feeds to those that exert major effects leads to substantial savings in time and cost [10, 11]. Screening experimental designs with these components at low and high ranges of concentration is helpful for determining which components have important effects on productivity. After determining the critical components, optimization of their amount is necessary. This step can be performed with DOE methods, such as response surface methodology (RSM). Amino acids are amongst the most basic nutrients for promoting cell growth and progress in productivity. They are nitrogen sources and the building blocks of proteins as well as mediators of numerous metabolic pathways [3, 12–14]. Although amino acid supplementation is recognized as one of the vital components in cell culture medium design and optimization, only a few reports have been focused on it [3, 8, 12–17].

In this study, we determined the critical amino acids for enhancement the target mAb production in a specific CHO cell line using Plackett-Burman design. After finding the critical amino acids, the concentration of these components in feed was optimized by RSM using a Box-Bencken design. The best group from RSM analysis was selected to explore the effect of designed amino acid feed on the main quality attributes of bevacizumab. N-glycan profiles, charge variant distribution, and low molecular weight forms are the main quality attributes for a monoclonal antibody and significant changes in these properties impact the mAb efficacy [18, 19]. The mentioned attributes are dependent in host cell line, clone, process conditions, and media composition. Therefore, analysing these product quality characteristics in process development is very useful to match the desired quality target product profile [20]. To our knowledge, this is the first report using the Plackett-Burman experimental design and RSM to investigate the most effective amino acids and optimize them in feed in order to enhance the recombinant mAb production. These strategy could be used for the optimization of other feed components (i.e., sugar, lipids, vitamins, and trace elements) to produce more developed feeds.

## Materials and Methods

### Cell line and culture media

Recombinant CHO cell producing bevasizumab, a mAb against vascular endothelial growth factor A, was kindly provided by Aryogen Biopharma (Alborz, Iran). The original source of CHO DG44 cell line was purchased from gibco (Catalog no: A10971-01). The basal culture medium was CDM4CHO (Hyclone laboratories, Utah, USA) which was supplemented with 6mM L-glutamine (Lonza, Verviers, Belgium) at the moment of preparation in accordance to the recommendations from the supplier.

### Culture conditions

CHO cells were cultured in a 500ml shaker flask with an effective volume of 100 ml, incubated at 37°C with a 5% solution of CO2, and agitated at 80 rpm. In the middle of logarithmic phase, the temperature shifted to 32°C. Each shaker flask was inoculated with an approximate cell density of 5×10^5^ cell/ml.

The amino acid feeds were added as multiple discrete additions to cultures on day 3, 5 and 7. All amino acid powders were purchased from HiMedia Laboratories Company (Mumbai, India).

### Cell quantification

Cell density and cell viability were determined by the Trypan blue exclusion method using a Neubauer cytometer. The harvested cells were centrifuged at 4000 rpm for 5 min and the supernatants were stored at -70°C for later analysis.

### Amino acid quantification

For quantification of amino acids, they were derivatized by OPA (Pickering Laboratories, CA, USA), separated by HPLC and detected by a florescence detector [21].

Consumption rates for each amino acid were determined from the analysis of the concentration profiles at the time observed in the 500 ml shaker flask. For that purpose, the consumption rates were assumed to follow the simple model d[AA]/dt = -qAA, where [AA] is a particular amino acid concentration, t is time, d[AA]/dt denotes the derivative of a particular amino acid concentration with respect to time, and qAA is the consumption rate of that amino acid. Therefore, qAA for each amino acid was calculated from the slope of the linear portion of the corresponding plot of concentration versus time.

### Monoclonal antibody quantification

The final concentration of the monoclonal antibody in the samples was determined by the MAbPac protein A affinity column (Thermo scientific, CA, USA). A simple two-buffer system was used for column operation. The equilibration buffer or Buffer A (5% PBS, 0.15M sodium chloride (Merck, Darmstadt, Germany) and 5% acetonitryl (Merck, Darmstadt, Germany)) was adjusted to pH 7.5 with ortophosphoric acid. The elution buffer (Buffer B) was the same buffer, but was adjusted to pH 2.5 with orthophosphoric acid. The elution gradient was programmed from 0% to 100% of elution buffer B in approximately 5 min. The standard curve generated with the purified monoclonal IgG and the mAb quantification was performed based on this curve.

### Experimental design

#### Screening the significant amino acids

A 20-run Plackett–Burman design was performed to explore the effect of 19 amino acids. According to this design matrix, a total of 20 trials were performed at various combinations of ‘high’ (1) and ‘low’ (-1) values of the different amino acids (Table 1), the low values were the amount of amino acids in basal medium and high values were determined with some pre-experiments. The statistical design and analysis were performed using the Design-Expert^®^ software version7 (Stat-EaseInc.Minneapolis, Minnesota, USA). The confidence level for significance was 95% (p<0.05). Briefly, this experimental design is based on the first order polynomial model:

Y=A0+ ∑AiXi

Where Y is the response (final mAb titre and integral viable cell concentration (IVCC)), A0 is the constant, and Ai is the linear coefficient for the independent variable Xi. The effect of each amino acid on the measured response (Y) was determined by an analysis of variance (ANOVA) which was performed for the amino acids in the model; values of Prob> F of less than 0.05 indicated that the model terms were significant.

**Table 1:** Plackett–Burmandesign matrix, twenty runs were designed at various combinations of ‘high’ (1) and ‘low’ (-1) values of 19 different amino acids.

Run	Asp	Glu	Asn	Ser	His	Arg	Gly	Thr	Ala	Tyr	Met	Val	Phe	Iso	Leu	Lys	Cys	Trp	Pro
1	1	-1	-1	-1	-1	1	1	-1	1	1	-1	-1	1	1	1	1	-1	1	-1
2	1	1	-1	1	1	-1	-1	1	1	1	1	-1	1	-1	1	-1	-1	-1	-1
3	-1	1	1	-1	-1	1	1	1	1	-1	1	-1	1	-1	-1	-1	-1	1	1
4	1	-1	-1	1	1	1	1	-1	1	-1	1	-1	-1	-1	-1	1	1	-1	1
5	-1	-1	-1	-1	-1	-1	-1	-1	-1	-1	-1	-1	-1	-1	-1	-1	-1	-1	-1
6	-1	1	-1	1	-1	-1	-1	-1	1	1	-1	1	1	-1	-1	1	1	1	1
7	1	-1	1	1	-1	-1	1	1	1	1	-1	1	-1	1	-1	-1	-1	-1	1
8	-1	1	1	1	1	-1	1	-1	1	-1	-1	-1	-1	1	1	-1	1	1	-1
9	-1	1	-1	-1	-1	-1	1	1	-1	1	1	-1	-1	1	1	1	1	-1	1
10	1	1	1	-1	1	-1	1	-1	-1	-1	-1	1	1	-1	1	1	-1	-1	1
11	1	1	1	1	-1	1	-1	1	-1	-1	-1	-1	1	1	-1	1	1	-1	-1
12	-1	-1	1	1	1	1	-1	1	-1	1	-1	-1	-1	-1	1	1	-1	1	1
13	1	-1	1	-1	-1	-1	-1	1	1	-1	1	1	-1	-1	1	1	1	1	-1
14	-1	-1	1	1	-1	1	1	-1	-1	1	1	1	1	-1	1	-1	1	-1	-1
15	1	1	-1	-1	1	1	1	1	-1	1	-1	1	-1	-1	-1	-1	1	1	-1
16	-1	-1	-1	1	1	-1	1	1	-1	-1	1	1	1	1	-1	1	-1	1	-1
17	-1	-1	-1	-1	1	1	-1	1	1	-1	-1	1	1	1	1	-1	1	-1	1
18	-1	1	1	-1	1	1	-1	-1	1	1	1	1	-1	1	-1	1	-1	-1	-1
19	1	-1	1	-1	1	-1	-1	-1	-1	1	1	-1	1	1	-1	-1	1	1	1
20	1	1	-1	1	-1	1	-1	-1	-1	-1	1	1	-1	1	1	-1	-1	1	1

#### Locating optimum concentration of significant amino acids

After the selection of four significant amino acids by Plackett-Burman design, Box–Behnken statistical experimental design (a common method of RSM) was employed for the further optimization of these amino acids based on 29 sets of experiments. The levels corresponding to the center and the walls of this design were designated 0, –1 and +1. Table 2 lists various assemblies that were used in the design. The low level used in the design (designated –1) was the same as the higher level used in the Plackett-Burman design. The middle and high levels (designated 0 and +1) chosen in the Box-Bencken design were respectively 25% and 50% higher than the low levels. The final mAb titre was used as the response parameter in this step.

**Table 2:** Box-Bencken design matrix, the low level used in the design (–1) was the same as the higher level used in the Plackett-Burman design. The middle and high levels (0 and +1) were respectively 25% and 50% higher than the low levels.

Run	Asp	Glu	Arg	Gly
1	0	0	1	-1
2	1	0	0	-1
3	0	-1	1	0
4	0	-1	-1	0
5	0	1	-1	0
6	-1	-1	0	0
7	0	-1	0	-1
8	-1	0	0	-1
9	0	1	0	1
10	1	0	1	0
11	-1	1	0	0
12	1	1	0	0
13	0	0	0	0
14	-1	0	0	1
15	0	0	0	0
16	0	0	0	0
17	-1	0	1	0
18	-1	0	-1	0
19	1	0	0	1
20	0	-1	0	1
21	0	0	1	1
22	0	0	0	0
23	0	1	0	-1
24	0	0	0	0
25	1	0	-1	0
26	0	0	-1	1
27	0	1	1	0
28	1	-1	0	0
29	0	0	-1	-1

### Glycoprofiling of IgG

Ig Gglycoprofiling was carried out using N-Glycanase^®^ Kit (Prozyme, CA, USA) for quantifying the total of each N-glycans. Digestion and labelling of N-glycans from IgG was performed according to the manufacturer’s instructions. Labelled glycans were buffered in 70% v/v aqueous acetonitrile prior to the HPLC analysis using HPLC system equipped with fluorescence detector (KNAUER) and TSK-gel Amide-80, 4.6× 230 mm column (TOSOH biosience, Stuttgart, Germany). The mobile phases used were solvent A (100% acetonitrile) and solvent B (0.25M ammonium formate (Sigma- Aldrich, MO, USA) with pH = 4.4). Elution of the sample was performed using the following flow rate and gradient: T_0 min_ = 30% B with flow rate = 0.4mL/min, T_100_ min = 40% B with flow rate = 0.4 mL/min, T_103 min_ = 100% B with flowrate 1 mL/min, T113 min 100% B with flow rate 1 mL/min,T114 min 30% B with flow rate 1mL/min, T119 min 30% B with flow rate 1 mL/min, T120 min 30% B with flowrate 0.4 mL/min. Fluorescence detection was performed at Ex = 360 nm and Em = 425 nm. Analysis of the chromatogram was performed with ChromGate software (KNAUER). Relative quantification was performed using peak area and peak assignment based on retention time of known standards.

### Charge heterogeneity profiling of IgG

The IEX chromatography was performed on a liquid chromatograph (KNAUER,) Detection was performed at 280 nm. Flow rate was 0.8 mL/min, the injection volume was 80μL and the column compartment temperature was set at 25°C. Instrument control and data acquisition were performed using ChromGate software. The monolithic IEX column used was ProPac WCX-10, 4 mm × 250 mm (Thermo Scientific, USA). The mobile phases used were mobile phase A (0.01 M Sodium phosphate (Merck, Darmstadt, Germany) buffer pH 6.6) and mobile phase B (0.01 M Sodium phosphate buffer + 0.1M Sodium chloride pH 6.6). The elution was performed by an ascending gradient from 30% to 100% eluent B followed by isocratic elution for 2 min before returning the eluent composition to the starting condition (100% eluent A).

### Capillary electrophoresis SDS analysis

An Agilent 7100 CE instrument equipped with a photodiode array (PDA) detector (Agilent) was used to perform CE–SDS analyses. For the separation, fused-silica capillaries of 50 μm inner diameter with a total length of 30.2 cm and an effective length of 20 cm was used. The capillary was conditioned at 4 bar with 0.1 M NaOH, 0.1 M HCl, and deionized water for 3, 1, and 1 min, respectively, followed by filling the capillary with the running gel buffer for 10 min at 4bar. Sample loading was performed electrokinetically at 5 kV (reverse polarity) for 20 s. The sample analysis was performed by applying 15 kV (reverse polarity) for 30 min, and the detection wavelength was 220 nm. The capillary and autosampler were maintained at 25°C and 10°C respectively during the separation.

Samples were prepared by mixing 100 μg of protein with either 5 μl of 0.25M idoacetamide (GE Healthcare, Backing hamshire, Uk) for non-reduced samples or 5 μl of β-mercaptoethanol (Sigma Aldrich, MO, USA) for reduced samples and the total volume of the sample was then adjusted to 100 μl by adding an appropriate amount of SDS sample buffer (0.1M Tris–HCl (pH 9.0) and 1% SDS) in a microcentrifuge tube. The mixture was vortexed and centrifuged briefly to bring the contents down to the bottom of the tube. Samples were heated for 10 min at 70°C before analysis.

## Results and Discussion

### Amino acid analysis

The consumption rate for each amino acid can be calculated as d[AA]/dt = -qAA. The qAA for each amino acid can be calculated from the slope of the linear section of the matching concentration versus time graph [22]. If amino acids are added at inappropriate times and concentrations, undesirable by-products accumulates and osmolarity increases [23]. The amino acid analysis was performed to determine the appropriate optimal time to add amino acid feeds to the basal medium. Based on this analysis, it was found that day 3 was a good starting point. Furthermore, through some pre-experiments the maximum concentration of amino acids for the Plackett-Burman design was determined (data not shown).

However, as reported, the particular effect of each amino acid changes across the different CHO cell lines and different products [3, 24].

### Plackett–Burman design

The Plackett–Burman design and its analysis were performed with the design-expert^®^ software. As presented in Table 3, the effect parameter is the change in the final mAb titre response as the factor (amino acid concentration) changes from its low (-1) level to its high (+1) level. The sum of square for a term is the sum of squared deviations from the mean due to the effect of the term. The contribution% is calculated by the total sum of squares (SS) and then dividing each individual SS by the total SS, multiplied by 100. When all terms have the same degrees of freedom, the contribution% can be used to determine which terms are larger contributors than others. According to Table 3, based on the contribution% and effect parameters, Asp, Glu, Arg and Gly have the highest positive effect on the final mAb titre response, and Asn, Ser and Phe have the highest negative effect.

**Table 3:** The effects of amino acids on the final mAb titre, the effect parameter is the change in the final mAb titre as the amino acid concentration changes from its low level to its high level. Sum of square is the sum of squared deviations from the mean due to the effect of the term. Contribution% determines the individual amino acid contribution to final mAb titre in comparison to others.

Term	Effect	SumSquare	Contribution%
Asp*	8.1	328.05	24.99
Glu*	3.9	76.05	5.79
Asn*	-9.9	490.05	37.34
Ser*	-5.10	130.05	9.91
His*	0.90	4.05	0.31
Arg*	2.90	42.05	3.2
Gly*	2.70	36.45	2.78
Thr	-0.3	0.45	0.034
Ala	-0.1	0.050	3.809E-003
Tyr*	1.90	18.05	1.38
Met*	2.10	22.05	3.68
Val*	-3.50	61.25	4.67
Phe*	-3.70	68.45	5.22
Iso*	-1.10	6.05	0.46
Leu	0.3	0.45	0.034
Lys*	-1.10	6.05	0.46
Cys*	1.30	8.45	0.64
Trp	0.1	0.05	3.809E-003
Pro*	-1.70	14.45	1.10

* Amino acids in bold with a contribution% greater than 0.1 were chosen for the model in ANOVA.

Table 4 represents the effects of amino acids on the IVCC response. The values of the effect, sum square and contribution% parameters are the same as those of parameters in Table 3. Based on the contribution% and effect parameters, Asp, Glu and Gly have the highest positive effect on the integral viable cell concentration response, and Asn, Val and Phe have the highest negative effect.

**Table 4:** The effects of amino acids on Integral Viable Cell Concentration, the effect parameter is the change in IVCC as the amino acid concentration changes from its low level to its high level. SumSquare is the sum of squared deviations from the mean of the effect parameter for each amino acid. Contribution% determines the individual amino acid contribution to IVCC response.

Term	Effect	SumSquare	Contribution%
Asp*	637.20	2.030E+003	20.66
Glu*	433.00	9.374E+005	9.54
Asn*	-794.40	3.155E+006	32.11
Ser	53.60	14364.80	0.15
His	37	6845.00	0.070
Arg	87.80	38544.20	0.39
Gly*	357.20	6.380E+005	6.49
Thr*	-131.60	86592.80	0.88
Ala*	197	1.940E+005	1.97
Tyr*	159.80	1.277E+005	1.30
Met*	299.0	4.470E+005	4.55
Val*	-561.60	1.691E+006	17.21
Phe*	-176.60	1.559E+005	1.59
Iso	-47.20	1113920	0.11
Leu	35.60	6336.80	0.064
Lys	64.60	20865.80	0.21
Cys*	217.60	2.367E+005	2.41
Trp	-76.40	29184.80	0.03
Pro	8.00	320.00	3.256E-003

* Amino acids in bold with a % of contribution greater than 0.4 were chosen for the model in ANOVA.

Table 5 summarizes the ANOVA results for the amino acids which were chosen based on the contribution% for the final mAb titre. Amino acids with % of contribution higher than 0.1 were chosen for ANOVA. P value Prob> F parameter should be less than 0.05 to be strongly significant. The significance of the proposed model for the final mAb titre response was indicated by the F-value (349.75) and a low probability value (P-value < 0.0001). The proposed model for the final mAb titre response is expressed as an empirical first order polynomial equation in terms of fifteen variables in Eq 1:

$$\begin{array}{l} \text{final mAb titre}=\;+52.85\;+4.05\text{A}+1.95\text{B }-4.95\text{C }-2.55\text{D }+0.45\text{E }+1.45\text{F }+1.35\text{G }+0.95\text{K}\\ +1.05\text{L }-1.75\text{M }-1.85\text{N }-0.55\text{O }-0.55\text{Q }+0.65\text{R}-0.85\text{T} \end{array}$$ (1)

**Table 5:** ANOVA results for Plackett-Burman analysis for the final mAb titre response

Source	Sum of square	df	Mean Square	F Value	P Value Prob> F
Final mAb titre					
Model	1311.55	15	87.44	349.75	<0.0001
Asp	328.05	1	328.05	1312.20	<0.0001
Glu	76.05	1	76.05	304.20	<0.0001
Asn	490.05	1	490.05	1960.20	<0.0001
Ser	130.05	1	130.05	520.20	<0.0001
His	4.05	1	4.05	16.20	0.0158
Arg	42.05	1	42.05	168.20	0.0002
Gly	36.45	1	36.45	145.80	0.0003
Tyr	18.05	1	18.05	72.20	0.0011
Met	22.05	1	22.05	88.20	0.0007
Val	61.25	1	61.25	245.00	<0.0001
Phe	68.45	1	68.45	273.80	<0.0001
Iso	6.05	1	6.05	24.20	0.0079
Lys	6.05	1	6.05	24.20	0.0079
Cys	8.45	1	8.45	33.80	0.0044
Pro	14.45	1	14.45	57.80	0.0016
Residual	1.00	1	0.25		
Cor Total	1312.55				

A, B, C, D, E, F, G, K, L, M, N, O, Q, R and T are coded values of Asp, Glu, Asn, Ser, His, Arg, Gly, Tyr, Met, Val, Phe, Iso, Lys, Cys and Pro, respectively.

Based on the results of Plackett-Burman analysis, the amino acids with the most pronounced effect on the final mAb titre were, in descending order by significance of effect, Asp, Asn, Ser, Glu, Phe, Val, Arg, Gly, Met, Tyr, Pro, Cys, Lys, Iso and His. For the range of concentrations which were tested in this experiment, Asn, Ser, Phe, Val, Iso and Lys were found to be harmful for the final mAb titre. However, the effects of Asp, Glu, Arg, Gly, Met, Tyr, Cys and His were positive. The other amino acids did not have a significant effect on the final mAb titre.

Table 6 summarizes the ANOVA results for the amino acids which were chosen based on the contribution% for the IVCC response. Amino acids with contribution% greater than 0.4 were selected for ANOVA analysis. As it was shown, the significance of the proposed model for the IVCC response was indicated by the F-value of 55.29 and a low probability value (P-value < 0.0001). The proposed model for the IVCC response is shown as an empirical first order polynomial equation in terms of eleven variables:

$$\begin{array}{l} \text{IVCC}=\;+3317.80+318.60\text{A }+216.50\text{B }-397.20\text{C }+178.60\text{G }-65.80\text{H }+98.50\text{J }+79.90\text{K }\\ +149.50\text{L }290.80\text{M }-88.30\text{N }+108.80\text{R} \end{array}$$ (2)

**Table 6:** ANOVA results for Plackett-Burman analysis for the IVCC response.

Source	Sum of square	df	Mean Square	F Value	P Value Prob> F
Integral Viable Cell Concentration (IVCC)					
Model	9.700E+006	11	8.818E+005	55.29	<0.0001
Asp	2.030E+006	1	2.030E+006	127.28	<0.0001
Glu	9.374E+005	1	9.374E+005	58.77	<0.0001
Asn	3.155E+006	1	3.155E+006	197.83	<0.0001
Gly	6.380E+005	1	6.380E+005	40.00	<0.0002
Thr	86592.80	1	86592.80	5.43	0.0482
Ala	1.940E+005	1	1.940E+005	12.17	0.0082
Tyr	1.277E+005	1	1.277E+005	8	0.0222
Met	4.470E+005	1	4.470E+005	28.03	0.0007
Val	1.691E+006	1	1.691E+006	106.04	<0.0001
Phe	1.559E+005	1	1.559E+005	9.78	0.0141
Cys	2.367E+005	1	2.367E+005	14.084	<0.0001
Residual	1.276E+005	8	15950.07		0.0049
Cor Total	9.828E+006	19			

Where A, B, C, G, H, J, K, L, M, N and R, are coded values of Asp, Glu, Asn, Gly, Thr, Ala, Tyr, Met, Val, Phe and Cys, respectively.

The amino acids with the highest effect on the IVCC were Asn, Asp, Val, Glu, Gly, Met, Cys, Ala, Phe, Tyr and Thr. Among them, Asn, Val, Phe and Thr were detrimental to the IVCC response and the effects of Asp, Glu, Gly, Met, Cys, Ala and Tyr were helpful for this response. As it was seen in this study, Asp, Glu and Gly had positive effect on both responses.

Table 7 lists the positive or negative effects of the amino acids on the final mAb titre. Based on these results, four amino acids (Asp, Glu, Arg and Gly) with the highest positive effects on the final mAb titre were chosen for the further optimization by RSM.

**Table 7:** The actual impacts of each significant amino acid on the final mAb titre, the actual impacts are the impacts of different amino acids on the final mAb titre and as it was expressed in Eq 1, these are the coefficients of related variables.

Amino acids	Actual impact
*Asp	4.05
*Glu	1.95
Asn	-4.95
Ser	-2.55
His	0.45
*Arg	1.45
*Gly	1.35
Tyr	0.95
Met	1.05
Val	-1.75
Phe	-1.85
Iso	-0.55
Lys	-0.55
Cys	0.65
Pro	-0.85

* Amino acids in bold were selected for further optimization by RSM.

### Response Surface Methodology

Asp, Glu, Arg, and Gly supplements were further optimized using response surface optimization. Table 2 presents various combinations used in this design. The amounts of the remaining amino acids in all assemblies were identical to those in the basal medium i.e. corresponding to –1 level in the Plackett-Burman design.

### Model fitting and analysis of variance (ANOVA)

The experimental results obtained under varying operational conditions were processed by the design expert software. The ANOVA results of the obtained model are summarized in Table 8.

**Table 8:** ANOVA results for RSM (Box-Bencken) analysis for the final mAb titre response.

Source	Sum of Squares	df	Mean Square	F Value	p-value Prob>F	
Model	843.54	20	42.18	22.08	<0.0001	significant
A-Asp	36	1	36	18.84	0.0025	
B-Glu	60.5	1	60.5	31.67	0.0005	
C-Arg	40.5	1	40.5	21.2	0.0017	
D-Gly	25	1	25	13.09	0.0068	
AB	20.25	1	20.25	10.6	0.0116	
AC	100	1	100	52.34	<0.0001	
AD	16	1	16	8.38	0.0201	
BC	9	1	9	4.71	0.0618	
BD	25	1	25	13.09	0.0068	
CD	25	1	25	13.09	0.0068	
A^2^	16.95	1	16.95	8.87	0.0176	
B^2^	8.09	1	8.09	4.23	0.0736	
C^2^	32.6	1	32.6	17.06	0.0033	
D^2^	19.68	1	19.68	10.3	0.0124	
A^2^B	24	1	24	12.56	0.0076	
A^2^C	140.17	1	140.17	73.37	<0.0001	
A^2^D	84.5	1	84.50	44.23	0.0002	
AB^2^	36.13	1	36.13	18.91	0.0025	
AC^2^	32	1	32	16.75	0.0035	
B^2^D	12.50	1	12.50	6.54	0.0338	
Residual	15.28	8	1.91			
Lack of fit	8.08	4	2.02	1.12	0.4567	Not significant
Pure error	7.20	4	1.80			
Core total	858.83	28				

The significance of the proposed model for the final mAb titre response was indicated by the F-value of 22.08 and a low probability value (P-value < 0.0001). The proposed model for the final mAb titre response is expressed as an empirical third order polynomial equation in terms of four variables (e.g. A, B, C and D; Eq 3):

$$\begin{array}{l} \text{final mAb titre }=\;+60.60\;+3.00\text{A }+2.75\text{B }+2.25\text{C }+2.50\text{D }-2.25\text{AB }+5.00\text{AC }+2.00\text{AD }\\ +1.50\text{BC }+2.50\text{BD }+2.50\text{CD }+1.62{\text{A}}^{\text{2}}+1.12{\text{B}}^{\text{2}}+2.24{\text{C}}^{\text{2}}+1.74{\text{D}}^{\text{2}}-3.00{\text{A}}^{\text{2}}\text{B }-7.25{\text{A}}^{\text{2}}\text{C }\\ +6.50{\text{ A}}^{\text{2}}\text{D }-4.25{\text{A B}}^{\text{2}}-4.00{\text{A C}}^{\text{2}}-2.50{\text{ B}}^{\text{2}}\text{D} \end{array}$$ (3)

Where A, B, C and D are coded values of Asp, Glu, Arg and Gly, respectively.

**Fig 1:**
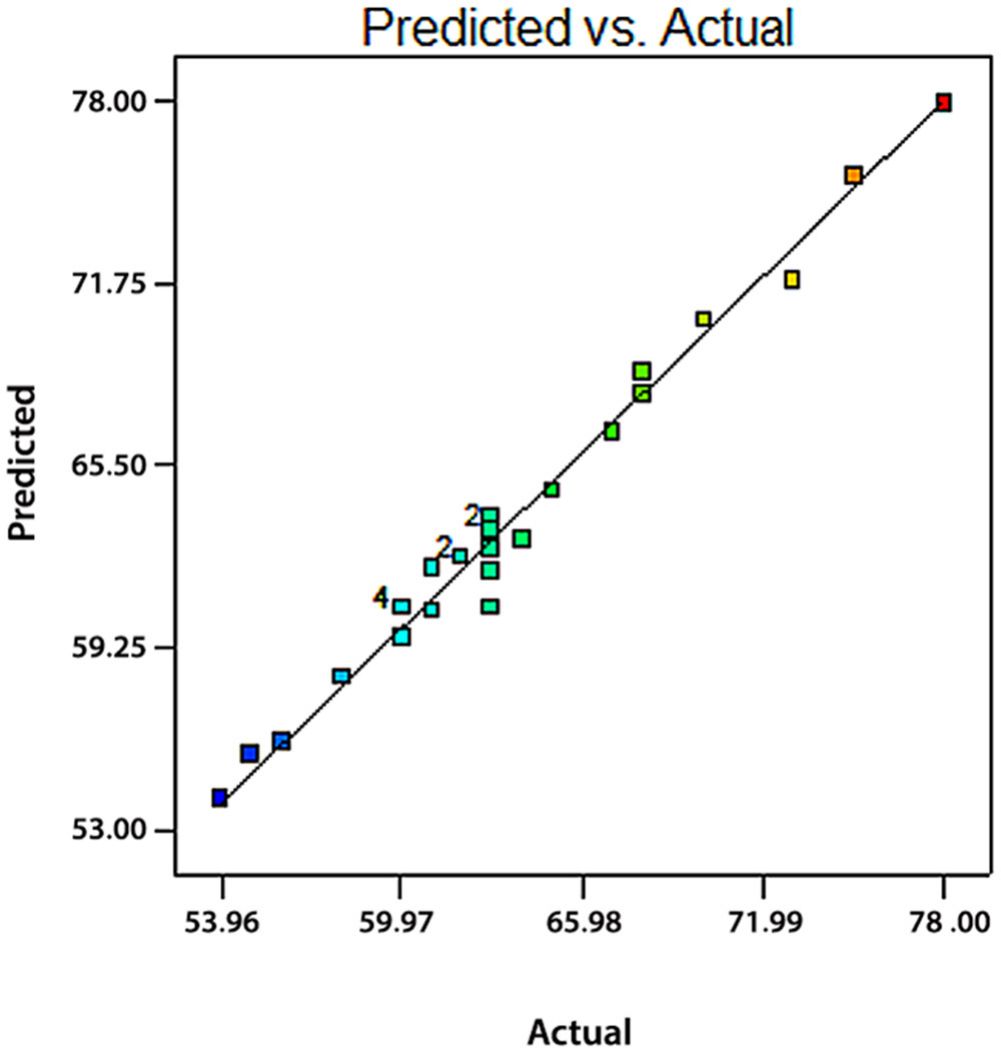
Predicted values versus actual values plot for the final mAb titre, It helps we detect a value, or group of values, that are not easily predicted by the model. The near 45 degree line of correlating plot shows the acceptable correlation between predicted and actual response values.

Fig 1 provides a graphical representation of the real final mAb titre versus the predicted values. It can be observed that there is a good agreement between experimental data and predicted results.

#### Response surface analysis

The response surface methodology (RSM) has several advantages, compared to the classical methods in which one variable at a time is used. Firstly, RSM gives a large amount of information from a small number of experiments. Indeed, classical methods are time consuming and a large number of experiments are required to explain the behaviour of a system. Secondly, in RSM it is possible to observe the interaction effect of the independent parameters on the response. The model equation easily clarifies effects for binary combination of the independent parameters [25].

**Fig 2:**
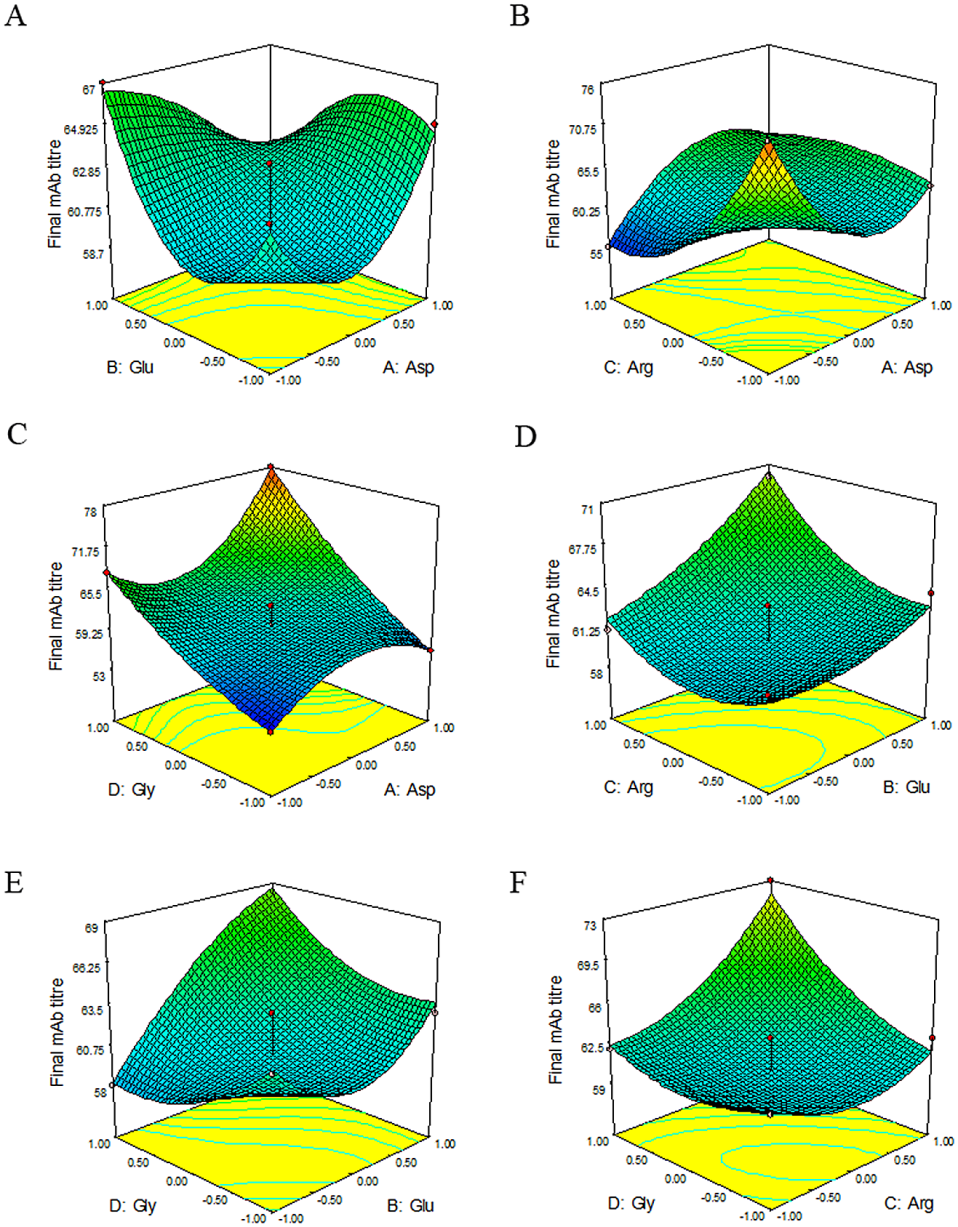
Representative charts of response surface. Response surface shows the effects and interactions of Glu and Asp, Arg and Asp, Gly and Asp, Arg and Glu, Gly and Glu, Gly and Arg on the final mAb titre response. (For instance in part B, if Asp and Arg concentrations become less or more than the middle point (0), the response will reach the maximum amount. These changes should be the same for both amino acids. It means Asp and Arg should increase or decrease in one direction and if both of them decrease, the response will be better. Also in part C, if Asp and Gly concentrations increase toward +1, the response will reach the maximum amount.)

The effects of selected amino acids and their interactions on the final mAb titre are graphically represented by three-dimensional response surface plots in Fig 2.

### The effects of amino acids

The addition of some amino acids may not always have a positive effect. For instance it was reported that for a CHO cell line producing t-PA, an increase in Asn concentration enhanced the production of ammonium. Furthermore, research suggests that a decrease in the intracellular concentration of some amino acids results in a reduction in t-PA production [12]. It is, therefore, very critical to distinguish the individual effects of the most important amino acids to be used for the feed. This is specific for each protein, cell line and each clone [24]. In this study, we showed that the use of Plackett–Burman and RSM statistical design is an appropriate strategy to screen the critical amino acids and optimize their concentrations in feed as to enhance the final mAb titre in a CHO cell line.

The suitable feed of the amino acids depends not only on the growth needs but also on the amino acid content of the expressed protein and this is individual for each cell line, each expressed protein and each medium which is used [3, 26].

**Fig 3:**
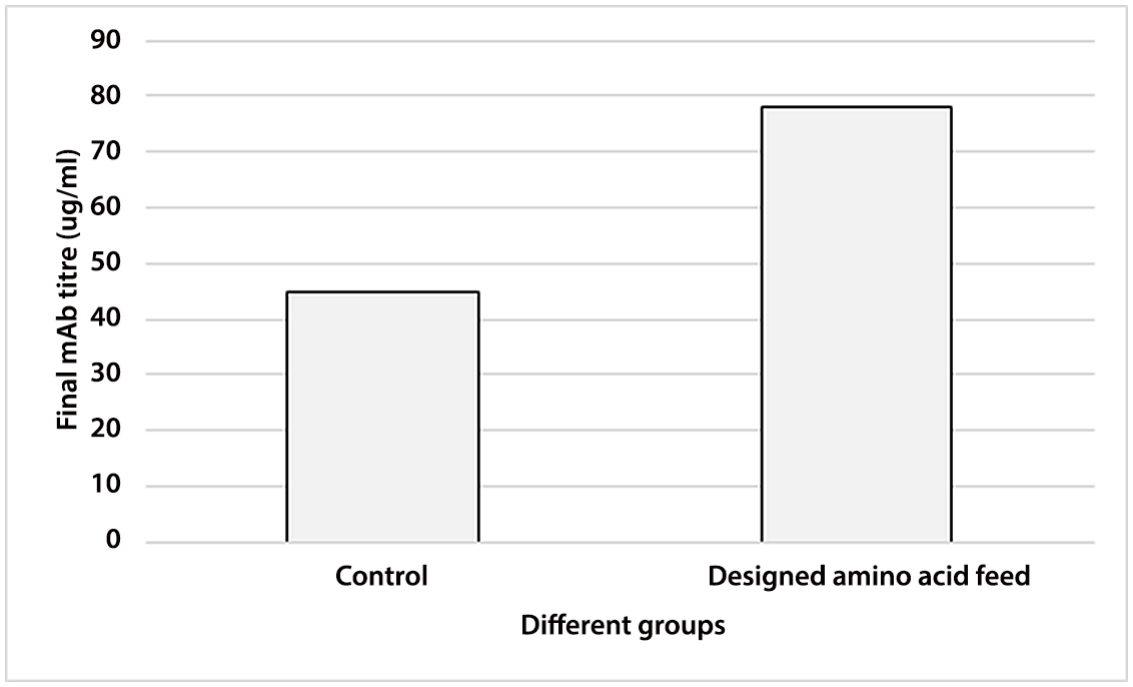
Comparison of final mAb titre in control and designed amino acid feed groups.

After the selection of more important amino acids by Plackett-Burman, their concentrations in feeds were further optimized by Box-Bencken. The optimized group increased the final mAb titre by 70%, compared to control group (Fig 3).

The selected amino acids were Tyr, Asp, Glu and Gly. Evidence has shown that if the amino acids have more mass fraction in target mAb, they will have more significant effect on the final mAb titre [3]. However, such effect was not observed in this study. So it seems that the role of significant amino acids in increasing the final mAb titre is related to their roles in different metabolic pathways. The selected amino acids in this study constitutes a moderate mass fraction in the structure of the produced mAb. Furthermore, according to the amino acid analysis results, the amount of Asp, Glu and Gly increased up to day 4, then the amount of Asp and Gly decreased and Glu concentration remained stable. It seems that these amino acids were necessary for the expression phase and the obtained results from the Plackett-Burman analysis confirmed that. It has been reported that Arg, Cys and Met are important for increasing the mammalian cell culture performance [13, 27]. In addition to the critical role of amino acids in mAbs structure, they play important role in metabolic pathways as mediators [3, 12]. Based on this role, the cell performance and subsequently mAb production are affected by amino acids. Therefore, the positive effect of Asp, Glu and Arg in our experiments seems to be related to their roles in TCA cycle. As an example, Asp, Glu and Arg entered the TCA cycle through oxaloacetate and α-ketoglutarate, which led to the production of more energy and to efficient cell metabolism [28]. William P.K. Chong et al. demonstrated that Asp from the culture medium contributed to the supply of malate in the cell and that over-expression of malate dehydrogenase II (MDH II) led to an increase in intracellular ATP and NADH, and improved integral viable cell number and target mAb titer [29].

Glutathione is a well-known indicator of oxidative stress [30], high levels of which have been related to high productivity [31]. Mitochondrial respiration is the main cause of reactive oxygen species (ROS) in the aerobic cell [32]. Thus, it has been hypothesized that high mAb producers experience increased aerobic metabolism that results in greater generation of ROS. This increases oxidative stress. Another important cause of ROS is derived from ER stress during protein folding [33]. It seems that high mAb producers have larger pools of GSH and GSSG to respond to these damaging effects than low mAb producers. GSH effectively neutralizes free radicals and other ROS species by being oxidized to GSSG [34]. Oxidative stress could be a possible reason of cellular apoptosis and growth limitation in the fed-batch cultures. Therefore, it appears that approaches to decrease this effect lead to cell growth, enhanced culture performance, and subsequently increase in productivity [30]. Glutathione biosynthesis is closely linked with some amino acids, such as Glu and Gly [31]. So the positive effect of Gly and Glu in production is probably related to their roles in glutathione pathway.

It should be emphasized that these results are cell line and clone specific and dependent on their metabolism. With regards to their metabolic properties, such as glucose consumption and lactate production, these results can be different. Also, the results are strongly dependent on the conditions applied in the experiments. For example, in the case of a specific CHO-DG44 cell line, Gonzalez et al. used a Plackett-Burman statistical design to assess the effect of the chosen amino acids on cell growth. They found that Leu and Arg had the highest negative and positive effects, respectively, on cell viability, while the addition of Leu and Thr resulted in the highest negative effects on growth rate. They further showed that Val and Arg had the highest negative impact on the final mAb titre [3]. In this study, we used Plackett-Burman to screen the amino acids in feed, but Gonzalez et al. used it to study the basal medium and added the amino acids as supplements at the beginning of culture. Amino acid needs are different for various cell lines [35]. Therefore, the amino acids identified as significant in the current study may or may not be important for other cell lines. The precise understanding of these events needs more studies.

### Glycoprofiling of IgG

Glycans play a very important role in biopharmaceuticals efficacy and their in vivo half-life [36, 37]. There are several reports on the effects of cell type, culture process, and culture media on the resulting glycoform profile [37]. Amino acid supplementation is critical for mammalian cell growth and productivity and their concentrations in media can cause changes in the glycosylation profile as well. Tho et al reported supplementing additional amino acids (cysteine, isoleucine, leucine, tryptophan, valine, asparagine, aspartic acid, and glutamate) that had been depleted in early culture, caused an increase in the lower sialylated fraction of recombinant human erythropoietin (rHuEPO) [38].

**Fig 4:**
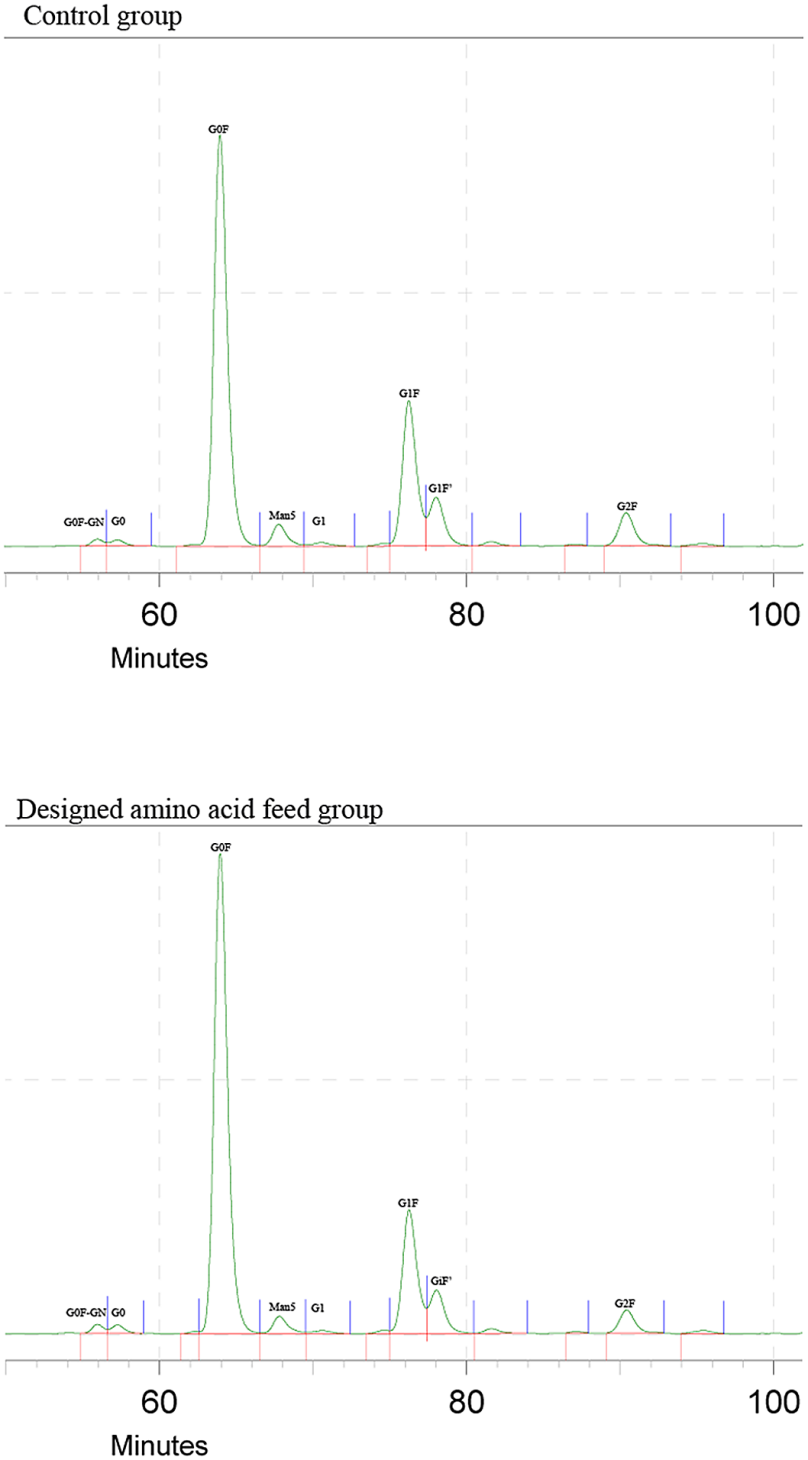
Glycoprofiling of bevacizumab in control and designed amino acid feed groups, glycan species abbreviations are as follow,G0: asialo, agalactose, biantennary complex (common core (Man3GlcNAc2) with terminal two GlcNAc residues), G0F: asialo, agalactose, biantennary complex, core substituted with fucose, G1: asialo, mono-galactosylated, biantennary complex, Man5: terminal two manose residues attached to the common core (Man3GlcNAc2), G1F/G1F’: asialo, mono-galactosylated, biantennary complex, core substituted with fucose, G2F: asialo, galactosylated, biantennary complex, core substituted with fucose.

In order to study the impact of the designed amino acid feed on the glycan profile of the product, glycoprofiling was performed for control and designed amino acid feed groups (Fig 4). Relative quantification was performed using peak area and peak assignment based on retention time of known standards.

The exposure of the terminal galactose residues in glycoproteins results in their rapid clearance from the circulation through asialoglycoprotein receptors on kupffer cells and reduce serum half-life [39, 40]. In this study, the N-glycan profile in designed amino acid feed group improved and the sum of all oligosaccharides without galactose increased in comparison to control group.

### Charge heterogeneity profiling of IgG

**Fig 5:**
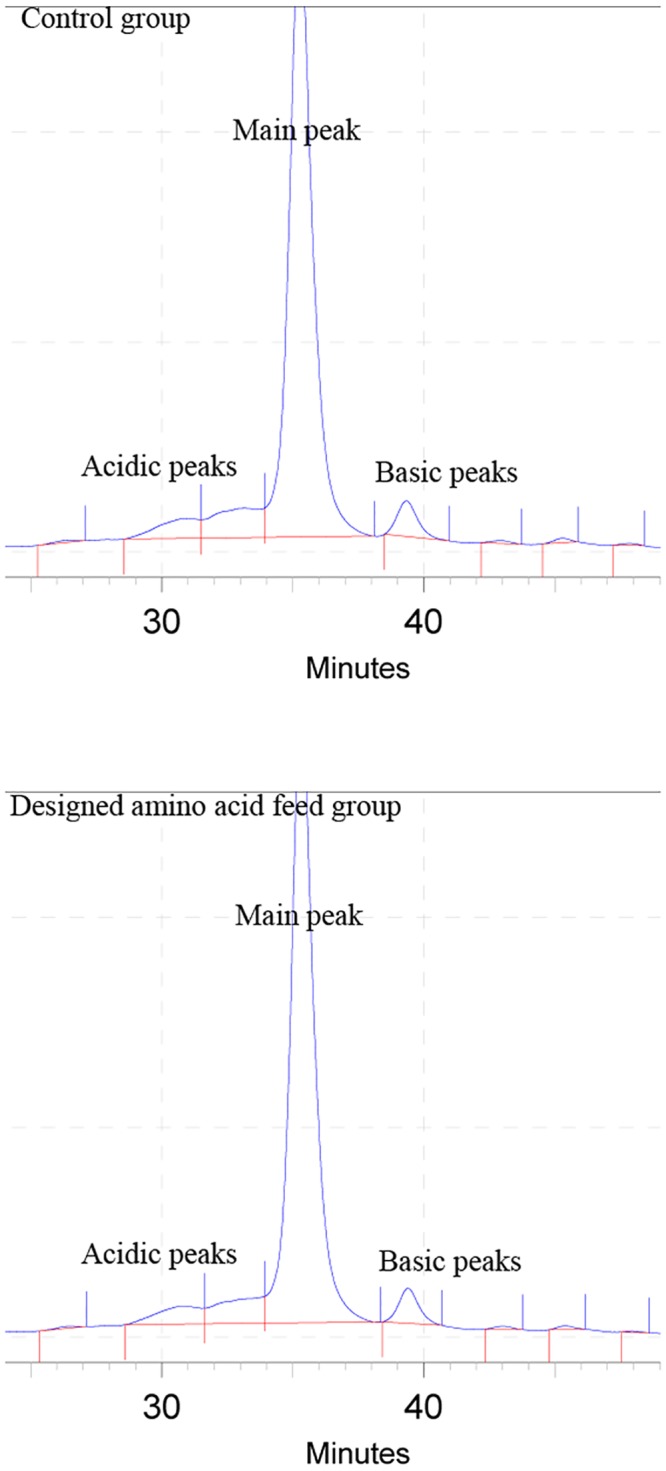
Charge heterogeneity profiling of bevacizumab in control and designed amino acid feed groups.

The impact of the designed amino acid feed on the charge variant distribution of bevacizumab was explored by liquid chromatography (Fig 5). There is no important difference between control and designed amino acid feed groups. The charge variant distribution of bevacizumab in both groups was similar.

### CE-SDS analysis

**Fig 6:**
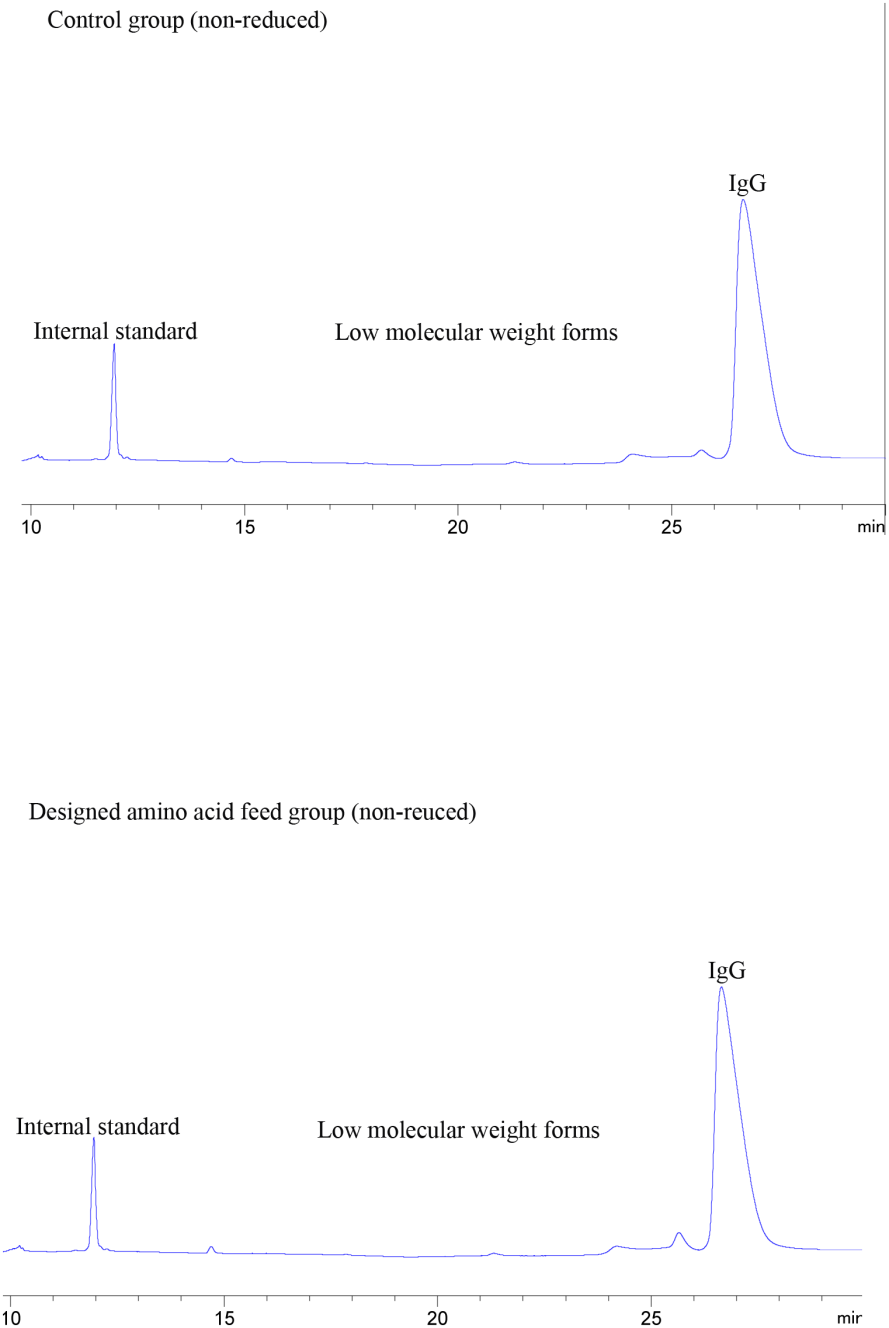
Electropherograms of non-reduced bevacizumab in control and designed amino acid feed groups.

**Fig 7:**
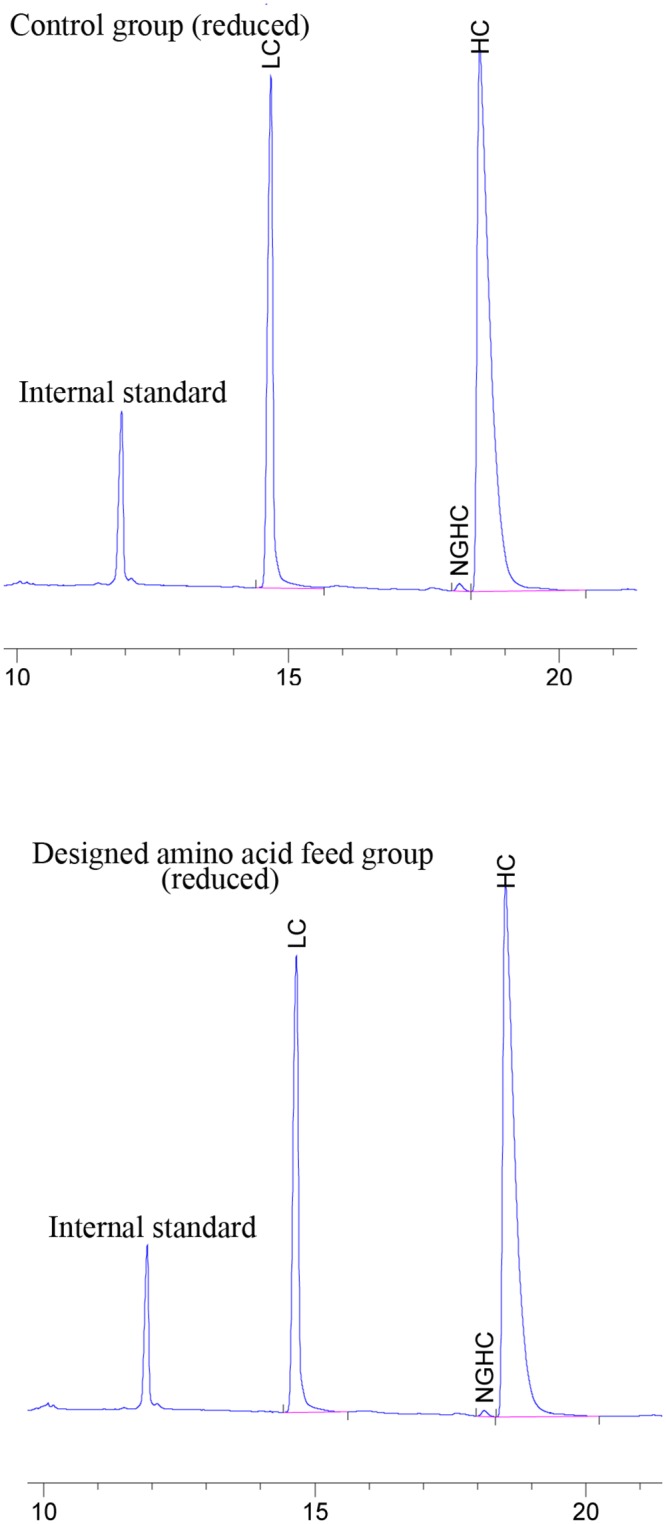
Electropherograms of reduced bevacizumab in control and designed amino acid feed groups, (LC: light chain, HC: heavy chain, NGHC: non glycosylated heavy chain).

To monitor the low molecular weight and non-glycosylated heavy chain (NGHC) forms, CE-SDS under non-reducing and reducing conditions was performed (Figs 6 and 7). The low molecular and NGHC forms amount were very similar for both groups.

## Conclusions

Focusing on amino acids, the current work suggested a logical method for determining and optimizing the components of cell culture feeds in a simple and rapid manner. As mentioned above, the particular effect of each amino acid changed between the different CHO cell lines and different products. To gain more insight into cellular mechanisms associated with different productivity, application of various omics technologies is needed.

Moreover, the use of experimental design methods, has become more common for screening and optimizing mammalian cell culture media and feeds, and supplies a logical strategy which can save time and resources.

## Supporting Information

S1 Fig Binding assay for control group. The assay was performed based on USP BEVACIZUMAB Summary Validation Report February 28, 2014. The results were analysed by PLA software.
(TIF)

S2 Fig Binding assay for designed amino acid feed group. The assay was performed based on USP BEVACIZUMAB Summary Validation Report February 28, 2014. The results were analysed by PLA software.
(TIF)
