# Supplementary figures and images for: Designed Amino Acid Feed in Improvement of Production and Quality Targets of a Therapeutic Monoclonal Antibody
Source: PLoS One. 2015 Oct 19;10(10):e0140597. doi: 10.1371/journal.pone.0140597 (PMC4610691; doi:10.1371/journal.pone.0140597)

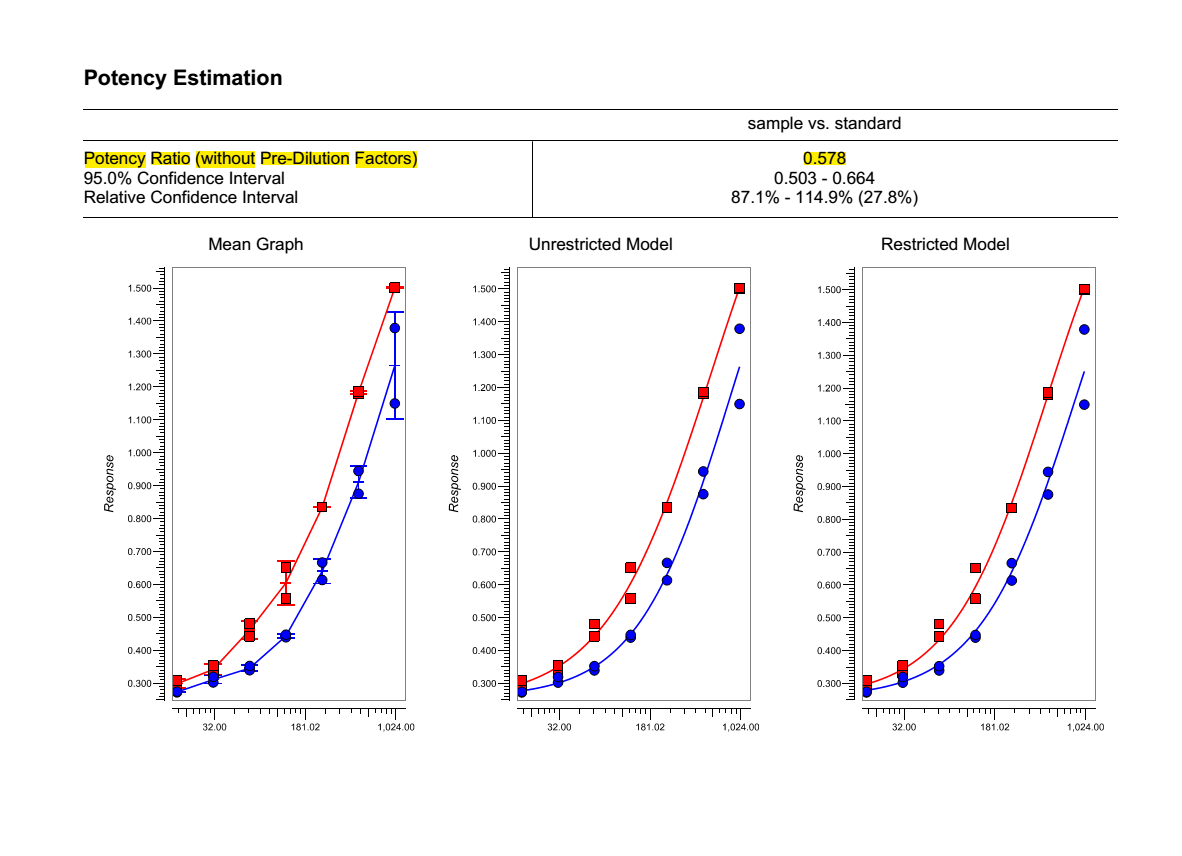

Supplement: S1 Fig — The assay was performed based on USP BEVACIZUMAB Summary Validation Report February 28, 2014. The results were analysed by PLA software. (TIF) [file pone.0140597.s001.tif]

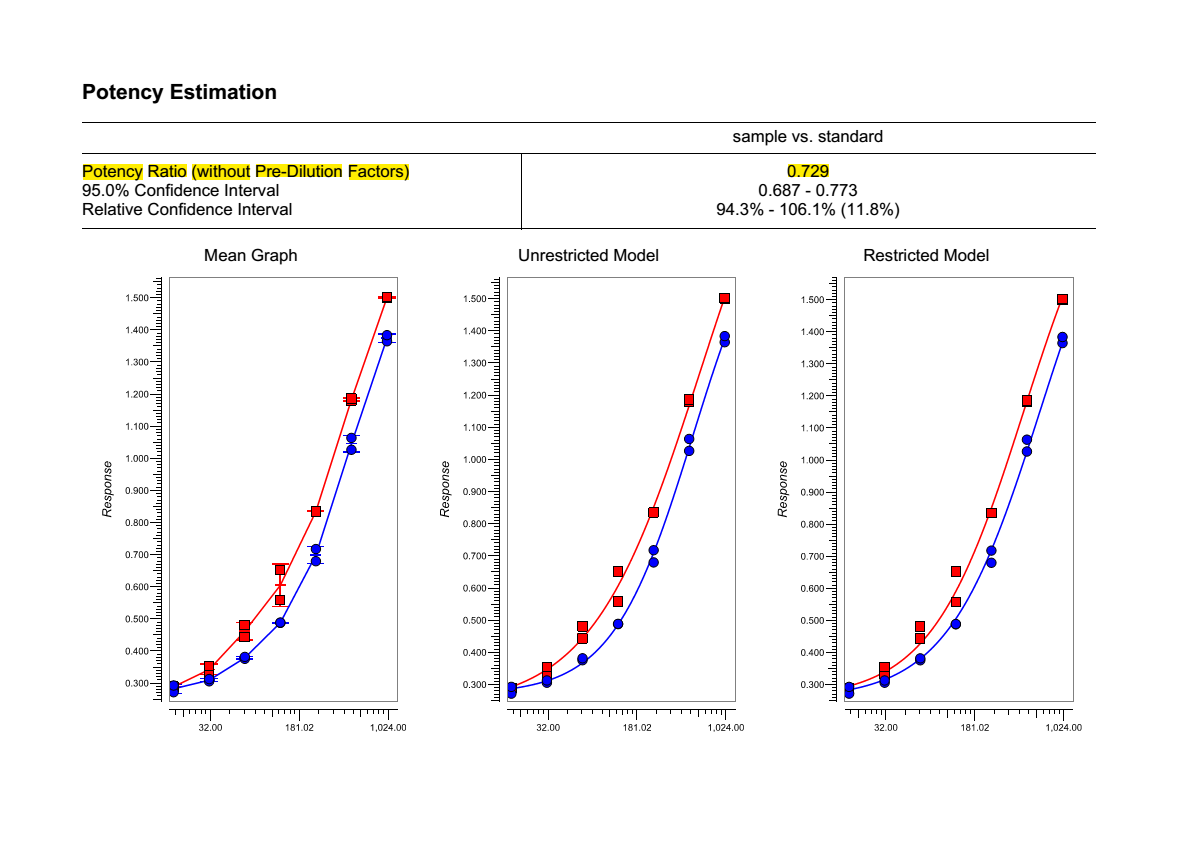

Supplement: S2 Fig — The assay was performed based on USP BEVACIZUMAB Summary Validation Report February 28, 2014. The results were analysed by PLA software. (TIF) [file pone.0140597.s002.tif]
